# miR-23b-3p Inhibits the Oncogenicity of Colon Adenocarcinoma by Directly Targeting NFE2L3

**DOI:** 10.1155/2021/8493225

**Published:** 2021-12-20

**Authors:** Guohong Huang, Yimei Yang, Mengxin Lv, Tian Huang, Xiaoyan Zhan, Yingjie Yao, Jianghou Hou

**Affiliations:** Clinical Research Center of Kunming Maternal and Child Health Hospital, Kunming 650031, China

## Abstract

**Background and Aims:**

MicroR-23b-3p (miR-23b-3p) has been found to be abnormally expressed in a variety of malignant tumors and to play a role in tumor inhibition or promotion. However, the regulatory mechanism of miR-23b-3p in COAD remains unclear. The purpose of this study was to investigate the clinical significance of miR-23b-3p expression in COAD cells and to explore its role and regulatory mechanism in the growth of COAD.

**Materials and Methods:**

Quantitative real-time polymerase chain reaction (qRT-PCR) was used to measure miR-23b-3p expression in COAD tissues and cell lines. After transfecting miR-23b-3p mimics into two human COAD cell lines (SW620 and LoVo), the cell counting kit-8 (CCK-8), colony formation, and 5-ethynyl-2′-deoxyuridine (EdU) assays were used to detect cell proliferation, the Transwell assay was used to measure cell migration and invasion capacity, and flow cytometry was used to evaluate cell apoptosis in vitro. In addition, a luciferase reporter assay was used to determine whether miR-23b-3p targets *NFE2L3*. The downstream regulatory mechanisms of miR-23b-3p action in COAD cells were also investigated. For in vivo tumorigenesis assay, COAD cells stably overexpressing miR-23b-3p were injected subcutaneously into the flank of nude mice to obtain tumors.

**Results:**

Significantly decreased expression of miR-23b-3p was detected in COAD tissues and cell lines. Exogenous miR-23b-3p expression inhibited cell proliferation, migration, and invasion and promoted cell apoptosis of COAD cells in vitro. Nuclear factor erythroid 2 like 3 (*NFE2L3*) was identified as a direct target gene of miR-23b-3p. In addition, reintroduction of *NFE2L3* partially abolished the anticancer effects of miR-23b-3p on COAD cells. Furthermore, miR-23b-3p overexpression hindered the growth of COAD cells in vivo.

**Conclusion:**

miR-23b-3p inhibited the oncogenicity of COAD cells in vitro and in vivo by directly targeting *NFE2L3*, suggesting the importance of the miR-23b-3p/NFE2L3 pathway in the development of COAD.

## 1. Introduction

Colon adenocarcinoma (COAD) is a common malignant tumor with high mortality rate [1]. According to the latest epidemiological statistics, colorectal cancer accounts for about 10% of new cancer cases worldwide, ranking third after lung cancer and female breast cancer [2]. In China, the incidence of colorectal cancer is on the rise, and with the increase of the age, the incidence of proximal colorectal adenocarcinoma increases significantly, while the proportion of rectal cancer decreases [3]. There is a tendency for the cancer to migrate to the proximal part. This study focuses on COAD.

Currently, the main methods for the clinical diagnosis of colon cancer include the fecal occult blood test, serum carcinoembryonic antigen detection, digital rectal examination, colonoscopy, and three-dimensional reconstruction of colon computed tomography (CT) [4, 5]. However, these methods have various disadvantages, such as low sensitivity, poor specificity, high cost, and invasiveness. Also, patients with mild symptoms and atypical colon cancer in the early and middle stages are easily missed or misdiagnosed [6]. Despite major advances in preventive screening, diagnosis, and treatment, the overall prognosis of COAD remains poor [7].

Molecular biology, particularly epigenetics, has been a hot issue in COAD prevention, early diagnosis, and prognosis in recent decades [8, 9]. Epigenetics was proposed as early as the 1950s to describe the interaction between genes and the environment during development, thereby regulating and determining the ultimate fate of tissues and organs, including histone modification and DNA methylation, DNA phosphorylation, and noncoding RNAs [10, 11]. The “noncoding RNA revolution” has witnessed the discovery of new types of RNA, which play key roles in a variety of diseases, including cancer [12]. MicroRNA (miRNA) is a type of noncoding regulatory RNA with a length of 18–24 nucleotides, which directly complements the 3′-UTR of mRNAs of target genes to inhibit their degradation or translation, thereby regulating their protein expression [13, 14]. One gene can be regulated by multiple miRNAs, and one miRNA can regulate the expression of more than a hundred genes. In fact, it is estimated that miRNAs can regulate about 30–80% of human genes [15].

Studies have shown that miRNAs are involved in numerous physiological and pathological processes, such as cell cycle regulation, differentiation, apoptosis, and invasion [16–18]. On the one hand, miRNAs can have a carcinogenic effect and promote tumor development by downregulating the expression of tumor suppressor genes. On the other hand, miRNAs can act as tumor suppressor genes and proto-oncogenes to inhibit tumor growth [19]. Many studies have shown that the expression of certain miRNAs in tumor tissues is dysregulated in a variety of solid cancer patients, which is closely associated with tumor development, invasion, metastasis, prognosis, and drug resistance [20].

In the initiation, development, invasion and metastasis of COAD, miRNAs have received increasing attention. Previous studies found that the expression level of miR-223 was significantly upregulated in COAD clinical samples and cell lines [21]. There was also a report that miR-1245a, miR-3682, miR-33b, and miR-5683 promoted the proliferation and migratory capacity of COAD cell, whereas miR-152 showed the opposite effects [22]. miR-23b-3p is dysregulated in several cancers and plays crucial roles in regulating cancer progression [23–29]. Chen et al. confirmed that miR-23b-3p level was consistently higher in serum samples from pancreatic cancer (PC) patients compared with those from healthy controls, and overexpression of miR-23b-3p promoted proliferation, migration, and invasion capability of PC cells in vitro [23]. A study by An et al. showed that miR-23b-3p inhibited autophagy mediated by ATG12 and HMGB2 and sensitized gastric cancer cells to chemotherapy [24].

However, little is known about the expression pattern and precise roles of miR-23b-3p in COAD. Therefore, in this study, we measured the expression of miR-23b-3p in COAD and investigated its clinical significance. More importantly, the precise roles and underlying molecular mechanisms of miR-23b-3p in COAD were elucidated.

## 2. Materials and Methods

### 2.1. Tissue Specimens

Surgically removed cancer tissue and normal colon tissue adjacent to cancer (normal mucosa 10 cm from the edge of the tumor) were collected from 23 matched pairs of COAD patients treated at the Clinical Research Center of Kunming Maternal and Child Health Hospital from October 2017 to October 2018. All specimens were diagnosed as COAD by two pathologists. The research was conducted in accordance with the Declaration of Helsinki. All patients had no history of other tumors, did not undergo radiotherapy, chemotherapy, or immunotherapy before surgery, and all signed informed consent. The Ethics Committee of the Kunming Maternal and Child Health Hospital reviewed and approved this study. The tissue was quickly (within 30 minutes after being isolated) placed in a liquid nitrogen tank for subsequent qRT-PCR analysis.

### 2.2. Analysis of miR-23b-3p and NFE2L3 Expression in the UALCAN Database

We analyzed and compared the expression of miR-23b-3p and NFE2L3 in COAD and normal tissues. In addition, UALCAN [30], which is a comprehensive and interactive web resource for analyzing cancer OMICS (TCGA, MET500, and CPTAC) data, was used to validate the results, especially by performing pan-cancer gene expression analysis. Using this public bioinformatics web tool, we were able to clearly determine the expression profiles of miR-23b-3p and NFE2L3 in human COAD tissues. Regarding how UALCAN databases were used, the detailed information is as follows. *Step 1.* Go to the analysis page of UALCAN (http://ualcan.path.uab.edu/index.html) and enter official symbol of gene (s) (such as miR-23b-3p, NFE2L3) in the text area. *Step 2.* Choose the TCGA dataset of interest from the drop-down menu and click “Explore” button to submit. *Step 3.* Output page provides links to analysis results and external database links.

### 2.3. Cell Lines and Culture Conditions

The human epithelial COAD cell lines SW620, SW1116, CW-2, and LoVo and the normal human colonic epithelial cell NCM460 were provided by the Cell Bank of the Chinese Academy of Sciences Type Culture Collection (Shanghai, China). Cells were cultured in Dulbecco's Modified Medium (DMEM) (Gibco/Invitrogen, Carlsbad, CA, USA) containing 100 U/ml penicillin/streptomycin and 10% fetal bovine serum (FBS). Cells were cultured in a humidified incubator containing 5% carbon dioxide and 37°C.

### 2.4. Cell Transfection

Lipofectamine 2000 (Invitrogen/Gibco) was used for transfection following the manufacturer's instructions. The COAD cell lines SW620 and LoVo in the logarithmic growth phase were digested with 0.25% trypsin, and then cells were seeded at 2 × 10^5^ cells/well in a six-well plate, cultured overnight. On the next day, after the cells grew into a monolayer, they were used for transfection experiments. The cells are grouped as follows: miR-23b-3p mimics group, miR-23b-3p NC group (NC), miR-23b-3p NC + pcDNA 3.1 NC group (NC + Ov-Ctrl), miR-23b-3p mimics + pcDNA 3.1 NC group (miR-23b-3p mimics + Ov-Ctrl), and miR-23b-3p mimics + pcDNA 3.1 NFE2L3 group (miR-23b-3p mimics + Ov-NFE2L3).

### 2.5. Quantitative Real-Time Polymerase Chain Reaction (qRT-PCR)

Total RNA from tissues or cells was extracted using the Trizol method and reverse transcribed into cDNA. Then, quantitative PCR was performed using a SYBR® Green Real-Time PCR Master Mix. U6 small nuclear RNA and *β-actin* served as internal controls for miR-23b-3p and *NFE2L3* mRNA levels, respectively. The 2^−∆∆Cq^ method was used to calculate the relative gene expression. The primers used were the following: forward primer of miR-23b-3p is 5′-ACACTCCAGCTGGGAUCACAUUGCCAGGG-3′, reverse primer of miR-23b-3p is 5′-CTCAACTGGTGTCGTGGAGTCGGCAATTCAGTTGAGGGTAATCC-3'; forward primer of *NFE2L3* is 5′-AGCGAGGAGAATGGGGTACT-3′, reverse primer of NFE2L3 is 5′-GCCTCCCAGTCAGGTTTCTC-3'.

### 2.6. Cell Counting Kit-8 (CCK-8), Colony Formation, and 5-Ethynyl-2′-deoxyuridine (EdU) Proliferation Assays

Log phase COAD cells were collected and seeded in 96-well culture plates at a density of 6 × 10^4^ cells/ml. Cell growth was measured at 24, 48, and 72 h after transfection. After culturing for the indicated time, 20 *µ*L of CCK-8 solution (Solarbio, Beijing, China) was added to each well and cultured at 37°C for 2 h. The absorbance value of each well was determined by colorimetry at a wavelength of 450 nm on a microplate reader. The cell growth curve was plotted with the different time points on the horizontal axis and light absorption values on the vertical axis.

For colony formation assay, COAD cells with different expression levels of miR-23b-3p or NFE2L3 were seeded into 6-well plates and cultured for 10 days. Colonies were fixed with methanol, stained with 0.1% crystal violet, and counted.

For EdU assay, the prepared transfected SW1116 and LoVo cells were collected and resuspended in DMEM medium (10% FBS) at a concentration of 5 × 10^5^ cells/mL. After placing the cells into 12-well plates with 0.1 ml in each well and culturing for 12 h, 10 *µ*M EdU was added into each well, and the plate was incubated for 2 h at 37°C to allow EdU incorporation. Subsequently, the cells were fixed with 4% paraformaldehyde for 15 min and stained for 15 min with a ClickiT EdU Assay kit (Invitrogen/Thermo Fisher Scientific, Inc.).

### 2.7. Transwell Migration and Invasion Assays

For the migration assay, after adjusting the cell concentration of the cell suspension from each treatment group to 1 × 10^6^ cells/ml in serum-free medium, a 150-*µ*l aliquot of the cell suspension was added to the upper chamber, and 750 *µ*l of medium containing 10% of FBS was added to the lower chamber, and the plate was incubated at 37°C for 24 h. Afterwards, each Transwell chamber was removed, washed twice with phosphate-buffered saline (PBS), fixed in the cell fixation solution for 20 min, and stained with crystal violet (0.1%) for 10 min, the upper surface was wiped with a cotton ball, and the cells were observed under a microscope. The number of penetrating cells was counted and used to evaluate the cell invasion ability. The invasion assay was performed in the same manner as the migration test, with an additional precoated with Matrigel in the upper chamber.

### 2.8. Western Blot Analysis

Total protein was extracted using radioimmunoprecipitation assay (RIPA) lysis buffer (Solarbio), and protein concentration was measured by the Bicinchoninic Acid (BCA) Protein Assay Kit (Solarbio). Equal amounts of proteins were separated by 10% sodium dodecyl sulfate-polyacrylamide gel electrophoresis (SDS-PAGE) and transferred onto polyvinylidene difluoride (PVDF) membranes. Then, the membranes were blocked with 5% (w/v) nonfat milk in Tris-buffered saline containing 0.1% Tween 20 (TBST) at room temperature and incubated with a primary antibody against NFE2L3 (Proteintech, Rosemont, IL, USA) at 4°C overnight (diluted at 1 : 800). Subsequently, after washing with TBST, the membranes were incubated with horseradish peroxidase-conjugated secondary antibody (Proteintech) and visualized using an ECL chemiluminescence Kit (Servicebio, Wuhan, USA).

### 2.9. Dual-Luciferase Reporter System Assay

The putative targets of miR-23b-3p were predicted using three miRNA target prediction software, namely, TargetScan (http://www.targetscan.org/), miRanda (http://www.microrna.org), and miRDB (http://www.mirdb.org/) [31].

The putative miR-23b-3p binding sequences of the wild-type (WT) or mutant (MUT) of NFE2L3 3′-UTR were separately amplified and subcloned into psiCHECK 2 luciferase reporter vector. COAD cells were seeded in 96-well plates and cotransfected with either miR-23b-3p mimics or control mimics and the WT/MUT vector using the Lipofectamine 2000 reagent according to the manufacturer's instructions. At 48 h after transfection, the cells were harvested and normalized to luciferase activity using the dual-luciferase reporter assay system (Promega Corporation, Madison, WI, USA).

### 2.10. Tumorigenesis in Nude Mice

All animal experiments were approved by the Ethics Review Committee of Kunming Maternal and Child Health Hospital and were conducted in accordance with the Animal Protection Law of the People's Republic of China. Four-six-week-old BALB/c nude mice were purchased from Vital River Laboratories (Vital River, Beijing, China). A total of 1 × 10^7^ cells transfected with miR-23b-3p mimics or NC were subcutaneously injected into the rear flank of the nude mice. The tumor width and length were measured every seven days and the tumor volume was calculated. Two months later, all nude mice were executed, and the xenografts were dissected and weighed.

### 2.11. Statistical Analysis

All results were analyzed using the SPSS software (IBM Corporation, Armonk, NY, USA). Student's two-tailed *t*-test was used for comparison of two treatment groups. One-way analysis of variance (ANOVA) was performed to evaluate the differences among multiple groups. The association between *NFE2L3* mRNA and miR-23b-3p expression levels was analyzed by Spearman's correlation analysis. *P* < 0.05 indicates a statistically significant difference.

## 3. Results

### 3.1. Expression of miR-23b-3p Is Decreased in COAD Tissues and Cell Lines

**Figure 1:**
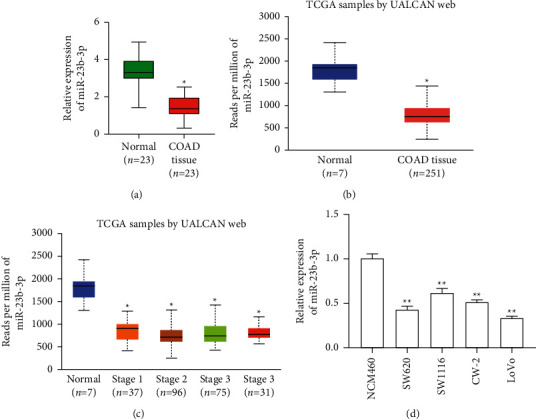
The expression of miR-23b-3p is decreased in COAD tissues and cell lines. (a) The expression of miR-23b-3p in 23 matched pairs of COAD tissues and adjacent normal colonic tissues was measured using qRT-PCR analysis. ^*∗*^*P* < 0.05 vs. normal colon tissues. (b, c) Analysis of the expression profiles of miR-23b-3p in COAD at different stages and normal colon tissues using the databases in the UALCAN platform. ^*∗*^*P* < 0.05 vs. normal colon tissues. (d) qRT-PCR analysis was performed to determine miR-23b-3p expression in four COAD cell lines (SW620, SW1116, CW-2, and LoVo). The normal colonic epithelial cell line NCM460 was used as a control. ^*∗*^*P* < 0.05 vs. NCM460. The results are expressed as the mean ± SD from three independent experiments.

qRT-PCR analysis was performed to determine the miR-23b-3p expression level in COAD tissues and adjacent normal colon tissues. The results showed that the expression level of miR-23b-3p was notably lower in COAD tissues (Figure 1(a)). Additionally, the analysis of the UALCAN database [26] also showed that the expression of miR-23b-3p is decreased in human COAD at different stages compared with normal colon tissues (Figures 1(b) and 1(c)). In addition, the data obtained from qRT-PCR analysis of the COAD cell lines revealed that miR-23b-3p was frequently downregulated in all four COAD cell lines (SW620, SW1116, CW-2, and LoVo) relative to its expression in normal colonic epithelial NCM460 cells (Figure 1(d)).

### 3.2. Overexpression of miR-23b-3p Inhibits the Malignant Phenotype of COAD In Vitro

**Figure 2:**
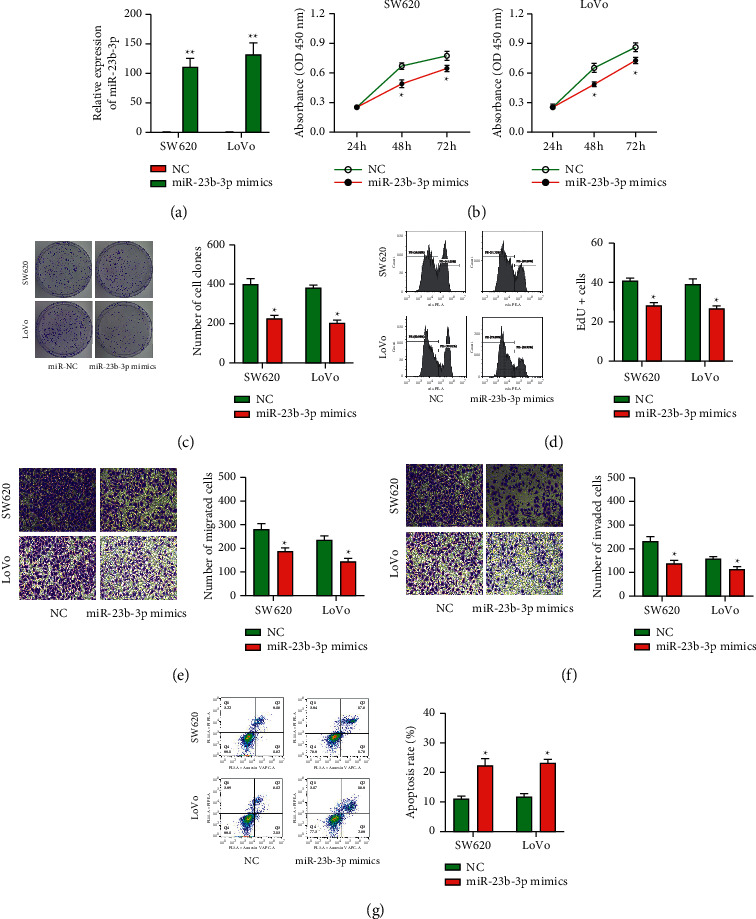
Overexpression of miR-23b-3p inhibits the proliferation, migration, and invasion of COAD cells. (a) Tumor cells were transfected with miR-23b-3p mimics or NC; qRT-PCR analysis was performed to determine transfection efficiency. (b–d) CCK-8, colony formation, and EdU assays were used to detect proliferation of COAD cells. (e, f) Transwell assay was performed to evaluate the migration and invasion capacity of COAD cells. (g) Flow cytometry was used to evaluate cell apoptosis. The results are presented expressed as the mean ± SD from three independent experiments. ^*∗*^*P* < 0.05 vs. NC.

In order to illustrate the functional roles of miR-23b-3p in COAD, we transfected miR-23b-3p mimics into two COAD cell lines, SW620 and LoVo, with low expression of miR-23b-3p. The results showed that the expression level of miR-23b-3p in the cells transfected with mimics was significantly increased by about 100–150 times (Figure 2(a)). The determination of the viability of these two cell lines in cell proliferation by the CCK-8, colony formation, and EdU assays indicated that miR-23b-3p overexpression significantly suppressed the proliferation of tumor cells (Figures 2(b)–2(d)). We also evaluated the effects of miR-23b-3p on the migration and invasion capacity of COAD cells. COAD cells overexpressing miR-23b-3p appear to have decreased migration capacity compared to the control cells (Figure 2(e)). Similarly, miR-23b-3p overexpression attenuated the invasion capacity of the COAD cells (Figure 2(f)). Furthermore, the analysis of cell apoptosis by flow cytometric revealed that cell apoptosis in the miR-23b-3p overexpression group was increased (Figure 2(g)).

### 3.3. NFE2L3 Is a Direct Target Gene of miR-23b-3p in COAD Cells

**Figure 3:**
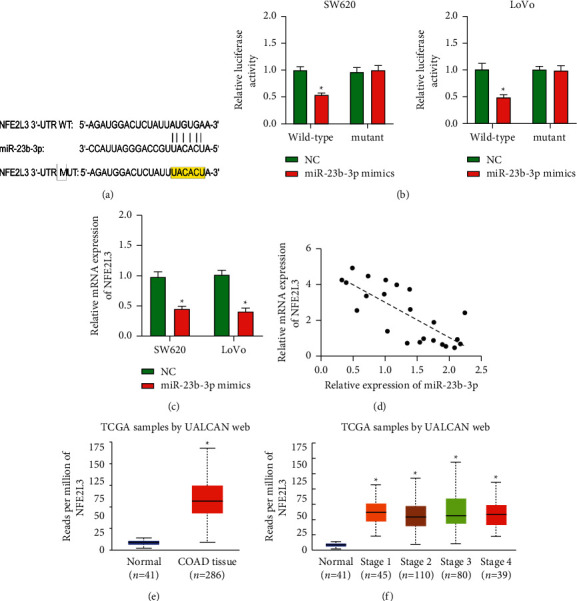
NFE2L3 is a direct target gene of miR-23b-3p. (a) The miRanda database showing the binding sites of miR-23b-3p with 3ʹ-UTR of NFE2L3 and the mutant sequence. (b) Luciferase reporter assay was used to determine whether the binding between miR-23b-3p and 3ʹ-UTR of *NFE2L3* affected the expression of *NFE2L*3. (c) The mRNA level of NFE2L3 was measured using qRT-PCR analysis after transfecting COAD cells with miR-23b-3p mimics or NC. ^*∗*^*P* < 0.05 vs. NC group. (d) Spearman's correlation analysis correlation between miR-23b-3p and *NFE2L3* mRNA levels in COAD tissues of Figure 1(a) (*n* = 23). (e, f) Expression profiles of NFE2L3 in COAD at different stages and normal colon tissues using the databases in the UALCAN platform. The results are expressed as the mean ± SD from three independent experiments. ^*∗*^*P* < 0.05 vs. normal colon tissues.

The search of putative target genes and binding site of miR-23b-3p by bioinformatics analysis revealed that *NFE2L3* is a target gene of miR-23b-3p (Figure 3(a)). A luciferase reporter assay was performed to determine whether miR-23b-3p was able to directly target the 3′-UTR of *NFE2L3* in SW620 and LoVo cells. The results revealed that miR-23b-3p mimics significantly decreased the relative luciferase activity of wild-type psiCHECK 2-NFE2L3-3ʹ-UTR, but its inhibitory effect was abrogated when the binding sequences of miR-23b-3p in the 3ʹ-UTR of NFE2L3 was mutated (Figure 3(b)). In addition, *NFE2L3* level was markedly decreased after transfecting COAD cells with miR-23b-3p mimics (Figure 3(c)). Furthermore, an inverse relationship between miR-23b-3p and *NFE2L3* mRNA levels was identified in COAD tissues (Figure 3(d)). Moreover, analysis of the UALCAN database showed that the expression of *NFE2L3* is increased in human COAD at different stages compared to normal colonic tissues (Figures 3(e) and 3(f)).

### 3.4. NFE2L3 Restoration Abrogates the Antitumor Effects of miR-23b-3p in COAD Cells

**Figure 4:**
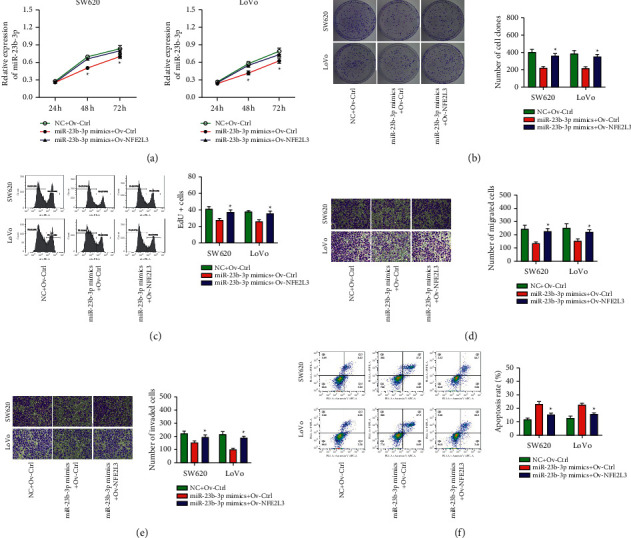
Restoration of NFE2L3 abrogates the antitumor effects of miR-23b-3p in COAD cell. MiR-23 b-3p mimics were cotransfected into LoVo cells with pcDNA 3.1-NFE2L3 or pcDNA 3.1. (a–c) The proliferation was determined using the CCK-8, cell colony formation, and EdU assays. (d, e) The migration and invasion were determined using the Transwell assay. (f) Cell apoptosis was determined using flow cytometry. The results are expressed as the mean ± SD from three independent experiments. ^*∗*^*P* < 0.05 miR-23b-3p mimics + Ov-NFE2L3 vs. miR-23b-3p mimics + Ov-Ctrl.

A series of rescue experiments were performed to further verify whether NFE2L3 contributes to miR-23b-3p-mediated tumor-suppressive activity in COAD cells. As expected, restoration of the NFE2L3 expression by further transfection of miR-23b-3p-overexpressing LoVo cells with the NFE2L3 overexpression vector pcDNA 3.1-NFE2L3 reversed the suppressive effects of miR-23b-3p overexpression on LoVo cell proliferation (Figures 4(a)–4(c)), migration (Figure 4(d)), invasion (Figure 4(e)), and cell apoptosis (Figure 4(f)) in vitro.

### 3.5. Overexpression of miR-23b-3p Hinders Tumor Growth of COAD Cells In Vivo

**Figure 5:**
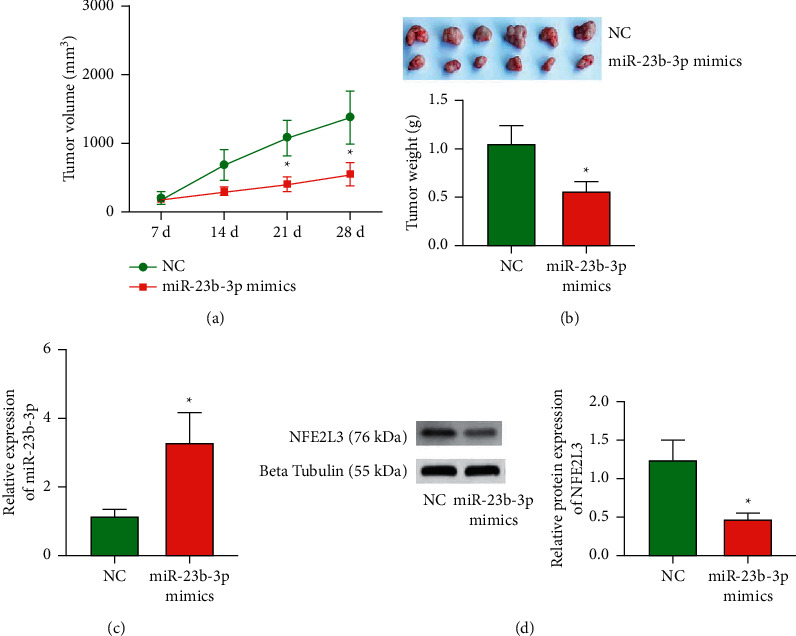
Expression of miR-23b-3p inhibits COAD growth in vivo. (a) Tumor volume was measured every seven days. (b) Tumors were isolated after 28 days of the injection of LoVo cells, and images of representative tumors were captured. The tumor volume and weight were calculated (*n* = 6). (c) The mRNA of miR-23b-3p in isolated tumors were measured by qRT-PCR analysis. (d) The expression of NFE2L3 was detected by Western blot analysis. ^*∗*^*P* < 0.05 vs. NC.

Tumor xenograft assay in nude mice was performed to evaluate the effect of miR-23b-3p overexpression on the growth of COAD cells in vivo. The tumor volume indicated an obvious suppression in the miR-23b-3p mimics group (Figure 5(a)). The tumor weights (Figure 5(b)) of the isolated tumor tissues from mice injected with miR-23b-3p mimics were significantly suppressed relative to those that received the NC. In addition, the xenograft had a higher miR-23b-3p level after injection of miR-23b-3p mimics (Figure 5(c)). Moreover, measurement of NFE2L3 protein expression by Western blot analysis showed signally decreased expression of NFE2L3 in the miR-23b-3p mimics group (Figure 5(d)).

## 4. Discussion

The initiation and development of tumors are the result of a variety of factors, and the mechanisms are complex. The conventional theory of tumor formation is that it begins with the division of a single mutated cell, whose offspring undergo sequential random genetic mutations that lead to somatic evolution, acquisition of neoplastic properties, and ultimately malignant phenotype involving multiple steps, genes, and pathways [32]. In recent years, this theory has been greatly challenged by findings of studies on epigenetics and stem cells. Researchers have found evidence that mutated, cancerous cells leading to early stages of tumors may develop directly from stem cells through epigenetic changes [33].

MiRNAs are an important component of epigenetics in a broad sense. They are endogenous single-stranded RNAs with regulatory function, which are widely distributed in eukaryotes. They can regulate protein expression by acting on mRNA. Proteins at all levels interact and coordinate with each other to form signal pathways, which participate in cell proliferation, differentiation, and apoptosis, to regulate the normal development of organisms, stem cell self-renewal, tissue repair, immune responses, and other normal physiological processes. Additionally, they also participate in the proliferation, apoptosis, invasion, metastasis, and chemotherapy resistance of tumor cells. Studies have shown that specific miRNAs can regulate the development of tumors in mouse models, and miRNAs can be combined with nanotechnology to treat tumor. Compared with other cancer treatment schemes, miRNAs have more stable antitumor effects and less adverse reactions, which opens a new a new chapter in cancer therapy based on miRNA [13–15].

Recently, miR-23b-3p has been specifically linked to various functions in various cancers through a cell-type-specific manner. In various kinds of tumors, miR-23b-3p was reported to act as a tumor suppressor [34, 35]. In this study, miR-23b-3p expression level was markedly reduced in COAD tissues compared with adjacent normal colonic tissues and significantly downregulated in all four COAD cell lines (SW620, SW1116, CW-2, and LoVo) analyzed relative to its expression in a normal colonic epithelial cell NCM460. The results suggested that miR-23b-3p may be involved in the development of COAD. The Cancer Genome Atlas (TCGA) database is a landmark cooperative project in the field of cancer research. As a result of its huge cancer gene database, TCGA improves the understanding of cancer pathogenesis and the basis of the clinical diagnosis and treatment of cancer. Analysis of TCGA data using the visualization software UALCAN [30] also showed that the expression of miR-23b-3p is decreased in human COAD at different stages compared with normal colon tissues. Experimentally, miR-23b-3p overexpression was found to inhibit the proliferation of COAD cells in vitro and decrease tumor growth and suppress COAD metastasis in vivo. These findings suggest that miR-23b-3p could be a potential diagnostic biomarker and therapeutic target for patients with COAD.

Determining the target gene of a miRNA allows us to understand its mechanism of action, which is at the core of research on miRNA. Accordingly, we conducted a preliminary prediction of the relationship between miR-23b-3p and *NFE2L3* through the TargetScan and miRbase databases. Then, we performed a dual-luciferase reporter assay detection using a 3′-UTR wild-type plasmid and a mutant plasmid construct of *NFE2L3*. The results showed that miR-23b-3p mimics significantly decreased the relative luciferase activity of the wild-type psiCHECK 2-NFE2L3-3ʹ-UTR construct, whereas the inhibitory effect was abrogated when the sequence of the binding site of miR-23b-3p in the 3ʹ-UTR of *NFE2L3* was mutated. This finding indicates that miR-23b-3p can directly bind to the 3-'UTR region of *NFE2L3*. In addition, we also found that the expression level of *NFE2L3* was significantly downregulated in COAD cells transfected with miR-23b-3p mimics. Furthermore, an inverse relationship between miR-23b-3p and *NFE2L3* mRNA levels was found in COAD tissues. This result indicates that miR-23b-3p can regulate *NFE2L3* expression by binding to its 3′-UTR in COAD cells.

The nuclear transcription factor NFE2L3 belongs to the basic-region leucine zipper transcription factor superfamily and the Cap “*n*” Collar \(CNC) family of proteins, which also includes NFE2LI, NFE2L2, NFE2P45, and transcriptional repressors BACH1 and BACH2. Compared with its paralog NFE2L2, the research on the function and regulation of NFE2L3 protein has not received much attention to date [36]. Recent studies have revealed that NFE2L3 may be associated with cell differentiation, inflammation, and cell carcinogenesis [37, 38]. The mechanism may be related to the maintenance of cell homeostasis by NFE2L3 involving antioxidant response elements and electrophilic response elements to regulate downstream gene expression. Some studies have shown that NFE2L3 was increased in COAD. Bury et al. found that NFE2L3 levels were elevated in colon cancer patients and the silencing of *NFE2L3* decreased colon cancer cell proliferation [39]. Uddin MN et al. identified numerous significantly upregulated genes, such as *CTHRC1*, *NFE2L3*, *SULF1*, *SOX9*, *ENC1*, and *CCND1*, and significantly downregulated genes, such as *MYOT*, *ASPA*, *NEXMIF*, *ARHGEF37*, *BCL2*, and *PPARGC1A*, in colon tumor stroma *versus* colon normal stroma [40]. Thus, we analyzed the gene expression databases in the UALCAN platform [30], and the result showed that the expression of *NFE2L3* is increased in human COAD at different stages compared with normal colon tissues. Moreover, we undertook to investigate the underlying mechanisms by which miR-23b-3p might affect NFE2L3 expression in COAD cells. Restoration of NFE2L3 expression partially attenuated the suppression phenotype driven by miR-23b-3p upregulation. Additionally, miR-23b-3p could suppress the aggressive progression of COAD by directly targeting *NFE2L3*, and such downregulation of *NFE2L3* expression by miR-23b-3p was essential for the miR-23b-3p-induced antitumor roles in COAD.

COAD develops in a complex environment in the human body. The environment of cells in in vitro experiments is very different from that of COAD in vivo, indicating that in vitro experiments have certain limitations. Therefore, we used animal models to further study the effects of miR-23b-3p on COAD behavior in vivo. The results showed that, compared with the control group, the tumor growth rate was significantly reduced in mice implanted with LoVo cells overexpressing miR-23b-3p.

Our study bears some limitations. One weakness is that we cannot obtain a large enough number of clinical case specimens to fully evaluate the clinical abnormal expression of miR-23b-3p. In addition, our study does not include the longitudinal measurement of miR-23b-3p during patient treatment, such as chemotherapy or local regional treatment, and we cannot provide data to show whether the changes in miR-23b-3p reflect tumor treatment response.

## 5. Conclusion

In this study, we confirm that the downregulation of miR-23b-3p is a common phenomenon in COAD tissues and cell lines. In addition, increased miR-23b-3p expression can inhibit the progression of COAD in vitro and in vivo by directly targeting *NFE2L3*. These findings provide a new insight into COAD carcinogenesis and suggest that miR-23b-3p could represent a potential therapeutic target for this type of cancer.

## Data Availability

The data used to support the study are available from the corresponding author upon request.
